# Measuring the contribution of human mobility to malaria persistence

**DOI:** 10.1186/s12936-020-03474-4

**Published:** 2020-11-11

**Authors:** Marcelo F. C. Gomes, Cláudia T Codeço, Leonardo S. Bastos, Raquel M. Lana

**Affiliations:** Programa de Computação Científica, Fundação Oswaldo Cruz, Avenida Brasil, 4365, Manguinhos, 21040-900 Rio de Janeiro, Brazil

**Keywords:** Malaria, Household survey, Mobility, Commutation, Logistic model

## Abstract

**Background:**

To achieve malaria elimination, it is important to determine the role of human mobility in parasite transmission maintenance. The Alto Juruá basin (Brazil) exhibits one of the largest vivax and falciparum malaria prevalence in the Amazon. The goal of this study was to estimate the contribution of human commutes to malaria persistence in this region, using data from an origin-destination survey.

**Methods:**

Data from an origin-destination survey were used to describe the intensity and motivation for commutations between rural and urban areas in two Alto Juruá basin (Brazil) municipalities, Mâncio Lima and Rodrigues Alves. The relative time-person spent in each locality per household was estimated. A logistic model was developed to estimate the effect of commuting on the probability of contracting malaria for a certain residence zone inhabitant commuting to another zone.

**Results:**

The main results suggest that the assessed population is not very mobile. A total of $96\%$ households reported spending over $90\%$ of their annual person-hour in areas within the same residence zone. Study and work were the most prevalent commuting motivations, calculated at $40.5\%$ and $29.5\%$ respectively. Spending person-hours in urban Rodrigues Alves conferred relative protection to urban Mâncio Lima residents. The opposite effect was observed for those spending time in rural areas of both municipalities.

**Conclusion:**

Residence area is a stronger determinant for contracting malaria than commuting zones in the Alto Juruá region. As these municipalities are a hotspot for *Plasmodium* transmission, understanding the main local human fluxes is essential for planning control strategies, since the probability of contracting malaria is dependent on the transmission intensity of both the origin and the displacement area. The natural conditions for the circulation of certain pathogens, such as *Plasmodium* spp., combined with the Amazon human mobility pattern indicate the need for disease control perspective changes. Therefore, intersectoral public policies should become the basis for health mitigation actions.

## Background

In 2015, Brazil launched The Malaria Elimination Plan, in alignment with the 2030 Sustainable Development Agenda [1]. Achieving this goal will require a better understanding of local malaria dynamics in remaining transmission hotspots. In this regard, malaria burden in Brazil is concentrated in the Amazon Basin [2]. Transmission is spatially heterogeneous within the Amazon Basin [3, 4] with high malaria transmission pockets associated to fish farming in both rural and urban areas [5], the arrival of susceptible individuals in new rural settlements located in forest fringes and illegal activities, such as mining and logging [6, 7].

Malaria risk factors display different determinant levels, from individual to household levels [8–12]. At the individual level, immunity, genetic background, nutrition, work activities, adherence to preventive practices and travel history, are important exposure and disease determinants, whereas type of household construction, distance to mosquito breeding sites, household income source, preventive habits and customs are important household level determinants [6, 9]. At the eco-social (community) level, type of landscape, economic activities and human occupation and mobility are noted as important determinants [8, 13, 14].

The focus of this study is human mobility and its contribution to malaria persistence. Human movements, seasonal, circular, or linear, within and across borders, are considered strong drivers for malaria (re)introduction [13, 15]. In the Amazon region, mobility drivers include seasonal economic activities, the search for urban services and illegal commodity transportation, among others [4, 7, 16]. Commuting requires long hours in small boats or 4 × 4 vehicles to cross rivers or poorly maintained dirty roads. The cost of travelling often implies in staying away from home for days. Carrasco-Escobar et al. [8] reported that human mobility in Peru is an important malaria persistence determinant in riverine communities, while Wesolowski et al. [13] indicated that human mobility influences the risk for malaria importation into low transmission areas in Kenya. The same scenario is noted in the Amazon Basin, i.e. in the state of Tocantins, which is a low endemicity area maintained by case imports [2, 14].

An important malaria transmission site in the Brazilian Amazon is the Alto Juruá region, in the state of Acre [16]. *Plasmodium vivax* is the main local pathogen, accounting for $70\%$ of all reported cases, followed by *Plasmodium falciparum* [2]. In 2018, the annual parasite index (API) was of 121.7 positive exams per 1000 inhabitants for vivax malaria and an API of 30 for *P. falciparum*. Vivax malaria is considered a neglected tropical disease and its elimination is one of the 2030 Agenda goals [1].

Reis et al. [5] reported a strong correlation between a malaria incidence time series in six Northwest Acre municipalities (Cruzeiro do Sul, Mâncio Lima, Rodrigues Alves, Marechal Thaumaturgo, Porto Walter, and Tarauacá). All but Tarauacá are located in the Alto Juruá region. The first three are connected by a single paved road, while the others are only accessible by waterways. Reis et al. [5, 17] postulated that shared environmental and social drivers could explain the observed malaria synchronicity in this region, contributing to high disease receptivity and vulnerability.

A household survey conducted in 2015 in 40 localities throughout this region detected a malaria trend along urban-rural gradient [9]. Although malaria was not clustered in any specific region, the odds of a household having malaria-infected inhabitants increased significantly along the assessed urban-rural axis, from $30\%$ in urban households to ca. $65\%$ in riverine households. Lana et al. [9] also reported increased odds of contracting malaria in rural households accessible by roads in comparison to those accessible by river only. These estimations did not take into account the time spent by individuals away from their residential areas.

The goal of this study is to revisit this household survey to estimate the contribution of human commuting to malaria persistence in the Alto Juruá region. In this article, it is postulated that commutation between urban and rural areas is important to the maintenance of high regional malaria indices. Understanding malaria dynamics along urban-rural gradient is useful to increase intervention strategy precision, by directing efforts towards main risk groups and areas.

To achieve this, a probabilistic model to estimate mobility contribution to the probability of contracting malaria at the household level was proposed, by using data from an origin-destination questionnaire that was part of the aforementioned household survey. First, estimates concerning the relative time spent in each locality by each individual during one year were postulated, followed by estimations concerning commuting contribution to the probability of contracting malaria.

## Methods

### Study area

**Fig. 1:**
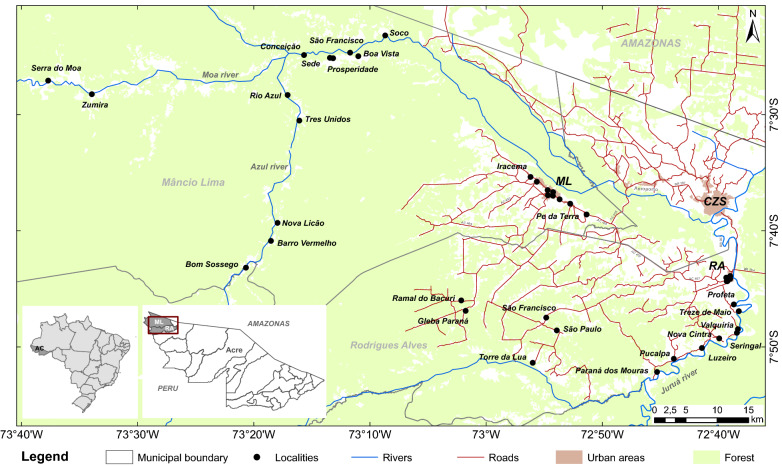
Map of the study area in the Alto Juruá region, state of Acre, Brazil. The black dots represent the surveyed communities. These communities are connected by dirt roads (red lines) and rivers (blue lines). Source: IBGE [18], PRODES [40], TerraClass [41], ANA [42] and the household survey described in Lana et al. [9]

The study area comprises a set of 40 urban and rural localities in two municipalities located in the Alto Juruá river basin, Acre, Brazil, Mâncio Lima (ML) and Rodrigues Alves (RA). These are predominantly rural and forested municipalities, inhabited by indigenous populations, traditional forest product extractivists, rural settlers and small-scale agriculture and fish farming businesses. The main administrative centre of the region, Cruzeiro do Sul, is located 12 km away from RA and 43 km from ML. Cruzeiro do Sul (CZS) is 700 km distant from Rio Branco, the state capital (Fig. 1).

ML ($7.5468^{\circ }$ S, $73.3709^{\circ }$ W) is the westernmost municipality of Brazil. Population density is low (2.79 inhabitants/km^2^), with $57.3\%$ of the 15,206 inhabitants living in the town and the remaining $42,7\%$ living in rural settlements scattered along dirt roads and along the Moa river and its effluents [18]. *Plasmodium vivax* is the most prevalent malaria parasite, with $API > 80$ every year since at least 2003. Malaria transmission in this municipality has being partially attributed to fish farming, which contributes to mosquito infestations, increasing risks for families practicing this activity [17]. The largest malaria activity peak was registered in 2005/2006, during a large malaria epidemic ($API = 505$), followed by a downwards trend after this date. In 2018, an increase in the number of malaria cases in Brazil was noted, attributed, in part, to the deterioration of malaria control programme [19]. During this period, ML experienced a surge in the number of vivax malaria cases, both in urban and rural settings ($API > 300$).

RA ($7.8819^{\circ }$ S, $73.3709^{\circ }$ W) is predominantly rural (4.68 inhabitants/km^2^). Only $30\%$ of the 14,389 inhabitants live in the town, which is located by the Juruá river. RA is small compared to ML, with less public services. Residents often commute to CZS, 12 km distant, in search of urban services. RA is located in a drier area, away from the flooded forest, and no fish farms are located within the town perimeter. Most of the population ($70\%$) lives in rural settlements scattered along a network of over 1000 km of dirt roads. Several riverine localities are accessible only by the Juruá river and its effluents during the rainy season [18]. As in ML, vivax malaria is the most prevalent, with an API always above 150 since 2004, with a peak noted in 2006 ($API = 994$) [2]. Urban malaria is less frequent in RA compared to ML, ranging from $API = 40$ to $API = 70$ most of the time, except during the 2006 epidemic, when API was 290. In contrast, malaria was more frequent in rural RA than in rural ML up to 2018 ($API > 189$), peaking at 1135 in 2006, decreasing to 227 only after 2018.

In the present study, data were compiled for four residence zones: MLu = urban Mâncio Lima, MLr = rural Mâncio Lima, RAu = urban Rodrigues Alves, RAr = rural Rodrigues Alves. Each residence locality belongs to only one of the four zones.

### Data

The Lana et al. 2015 survey assessed demographic and behaviour traits associated with self-reported malaria at the household level. Briefly, a total of 520 households were surveyed, distributed throughout 40 localities, as follows: nine in MLu ($n = 190$), five in RAu ($n = 102$), 13 in MLr ($n = 107$) and 13 in RAr ($n = 121$). The questionnaire was applied to a member of the household, who answered questions regarding him/herself and other members of the household. Information for a total of 2274 residents was obtained. Information on malaria was obtained with the question: “Have you had at least one episode of malaria in the last 12 months?”. A total of 233 ($44.8\%$) households reported at least one episode of malaria in the last 12 months, with $15.38\%$ in MLu, $10.77\%$ in MLr, $5.96\%$ in RAu, and $12.69\%$ in RAr [9].

One of the components of the survey was an origin-destination questionnaire. The member of the household responded how often he/she travelled to other localities to study, work, and other reasons, in the last 12 months. The time spent at each destination per year was recorded. After removing one household due to inaccurate information, the final data set for the analysis comprised 519 households.

### Mobility indicators

From the origin-destination data, the fraction of time spent at each destination was computed for each individual. To harmonize differences in time units that emerged between responses, whenever possible, the length of stay per trip was converted to hours and compiled for a period of 12 months, providing a measure of the total amount of hours per pair of origin-destination, per motive. In the case of pendular displacements for work and study, i.e. same-day round trips, standard work- and school -hours were applied, as described in more detail in the following paragraphs. Concerning a small set of responses (3/2274), the member of the household reported an area instead of a specific destination. A similarly small number of responses (6/2274) reported the locality but not the corresponding municipality. In these cases, the information based on the most likely destinations observed among neighbours was used.

#### Displacement for school attendance

 The time spent at a destination for schooling was estimated assuming standard school hours, $h_s = 4.5$ hours per day [20, 21] and $d_s = 200$ day-classes in a year [22], not accounting for extra time spent waiting for transportation or attending extra-curricular activities, among other activities.

Formally, the total amount of time spent at a given locality *l* by individual *i*, $t^{(s)}_{i,l}$, is given by: $t^{(s)}_{i,l} = h_s \times d_s \times {\mathscr {S}}_{i,l}$ where ${\mathscr {S}}$ is a matrix where ${\mathscr {S}}_{i,l}=1$ if the individual *i* attends classes at locality *l*, and 0 otherwise. If a household has $n_h$ cohabitants, with $n_s$ students, the total household person-time spent at each destination for studying will be $T^{(s)}_{h,l} = \sum _{i=1}^{n_h} t^{(s)}_{i,l}$.

#### Displacement for work

 Information on the average time spent and how frequently a household member travelled to a certain destination for work during the last 12 months was available for only a fraction of the responses. In these cases, the amount of time $t^{(w)}_{i,l}$ spent at the destination received the reported value. When the destination was mentioned but the time spent was absent, this information was imputed using the maximum working hours in Brazilian labour law, $h_w = 40$ hours per week. A total of 61/208 responses with only partial information regarding time spent were obtained, reporting total number of worked days but not how many hours per day, and another 45/208 reporting regular workdays. For the former, it was assumed 8 hours for each reported day. For the latter, it was assumed 5 days over 52 weeks, with 8 hours of work per day. The total person-time spent at each locality *l* by the $n_w$ workers in a household is $T^{(w)}_{h,l} = \sum _{i=1}^{n_h} t^{(w)}_{i,l}$, where $t^{(w)}_{i,l} = h_w \times d_w \times {\mathscr {S}}_{i,l}$ where ${\mathscr {S}}$ is a matrix where ${\mathscr {S}}_{i,l}=1$ if the individual *i* works at locality *l*, and 0 otherwise.

#### Total displacement

 The total time spent away from home to work or study was calculated on two spatial scales: First, the time spent in every locality *l* was counted, where locality was considered as any settlement or neighbourhood mentioned during the survey. Then, these localities were aggregated by residence zone (urban ML, rural ML, urban RA, rural RA, urban CZS, rural CZS and other). With this, local and regional mobility patterns can be assessed. Only commutations for work and study were considered in this calculation, since these are the main reasons for regular commuting.

The total person-time spent on each locality *l* by the cohabitants of household *h* is simply the sum of the time spent away for studying and for working, $T_{h,l}=T^{(s)}_{h,l}+T^{(w)}_{h,l}$. Complementary, it was assumed that the remaining time was spent at the residence locality:

1 $$\begin{aligned} T_{h,l[h]}&= n_h \times 24 \times 365 - \sum _{l \ne l[h]} \left( T^{(s)}_{h,l} + T^{(w)}_{h,l}\right) . \end{aligned}$$

Since households have different sizes, a normalized measure was computed by dividing $T_{h,l}$ by the total person-time for all household members (number of dwellers × 24 hs × 365 days):

2 $$\begin{aligned} \tau _{h,l}&= \frac{T_{h,l}}{n_h\times 24 \times 365}. \end{aligned}$$

The computation of residence zone level mobility was performed by replacing the person-hours spent at each locality *l* by the sum throughout localities belonging to the same zone *z*, $T_{h,z} = \sum _{l \in z} T_{h,l}$.

### Statistical model

A logistic model was fitted to the origin-destination data to estimate the effect of commuting on the probability of contracting malaria for a household member living in a residence zone *z* and commuting to a zone $z^{'}$. $Y_{h}$ is the number of people in a certain household *h* with $n_h$ cohabitants mentioning at least one episode of malaria in the past 12 months. If $\theta _h$ is the probability of a malaria case in the last 12 months at household *h*, $Y_h$ be modelled as a binomial process, dependent on the mobility pattern of its household members:

3 $$\begin{aligned} Y_{h}\sim & {} Bin(n_h, \theta _h), \qquad h=1,2,\cdots ,n=519, \end{aligned}$$

4 $$\begin{aligned} logit(\theta _h)= & {} \sum _{l} \beta _z \tau _{h,z}, \end{aligned}$$

with $\tau _{h,z}$ as given in Eq. (2) calculated per zone. The model has no intercept and each model coefficient $\beta _z$ can be interpreted as the probability of contracting malaria in the last 12 months when all people in a certain household spent their time at zone *z*.

Inferences for the model coefficients were performed under the Bayesian approach based on the approximation implemented in the R function *bayesglm* from the package arm [23]. In order to avoid numerical instability, relatively vague prior distributions were used for the model coefficients. These priors assume that the probability of contracting malaria in the absence of commutation is of approximately $12\%$ ($logit^{-1}(-2) \approx 0.119$) ranging from $1.8\%$ to $49.0\%$. This is in accordance with the average annual parasite index observed in the area in 2015. Mathematically,

5 $$\begin{aligned} \beta _z \sim N(-2, 1), \qquad \forall z. \end{aligned}$$

For any location *l*, the probability of a malaria case in the last 12 months is estimated as

6 $$\begin{aligned} \pi _l&= logit^{-1}\left( \sum _{z'} {\bar{\tau }}_{l,z'} \beta _{z'} \right) \nonumber \\&= \frac{ e^{\sum _{z'} {\bar{\tau }}_{l,z'} \beta _{z'}} }{ 1 + e^{\sum _{z'} {\bar{\tau }}_{l,z'} \beta _{z'}} }, \end{aligned}$$

where ${\bar{\tau }}_{l,z'}$ is a normalized average of the ratio of person-time spent at zone $z'$ by individuals from location *l*. That is

7 $$\begin{aligned} {\bar{\tau }}_{l,z'}&= \sum _{h \in l} \frac{T_{h,z'}}{n_l \times 24 \times 365}, \end{aligned}$$

where $n_l = \sum _{h \in l}n_h$ is the number of respondents in location *l*.

### Scenarios

The fitted model was used to calculate the expected probability of contracting malaria under different commuting scenarios. The baseline is zero mobility, that is, a scenario with nobody leaving their home. This baseline was compared with scenarios where individuals spent up to $50\%$ of their time in ML and RA rural or urban areas.

The calculated effect is obtained from the fitted model as the ratio $\tau _{z,z'}$ of person-hours spent at zone $z'$ for a resident of *z* resident, with the difference in malaria case probability calculated as:

8 $$\begin{aligned} \alpha _{z,z'} = logit^{-1}\left( \left( 1 - {\bar{\tau }}_{z,z'}\right) \beta _{z} + {\bar{\tau }}_{z,z'} \beta _{z'} \right) - logit^{-1}\left( \beta _z \right) . \end{aligned}$$

The first term is the estimated malaria case probability given the mobility pattern scenario, while the second term is the probability without leaving the residence zone (baseline). Therefore, it can be interpreted as the destination’s malaria probability contribution at origin. To take into account statistical uncertainties, 1000 samples were generated from the posterior distribution of each parameter $\beta _z$ obtained from the logistic model.

Along with R package arm [23] used for the aforementioned statistical model, the tidyverse [24] package was used for data processing, ggplot2 [25], and Inkscape [26] for plot and graph styling, the seriation [27] package for heatmap plot ordering, and cytoscape [28] for network representation.

## Results

### Mobility to areas outside the study region

As a whole, the participants reported 19 destinations outside the study area visited in the last 12 months, totaling 17 in Brazil, one in Peru and one in Bolivia. In Brazil, the following destinations were cited: nine in Acre, one in Rondônia, one in São Paulo, one in Rio de Janeiro, one in Rio Grande do Norte and four in Amazonas (see Additional file 1). Of the 519 households, only 92 reported at least one travel to localities outside the study area. Of those, only 10 households reported destinations outside the state of Acre. The main reasons for travelling to places outside the study area were for medical assistance and leisure activities.

### Mobility within the study region

**Fig. 2:**
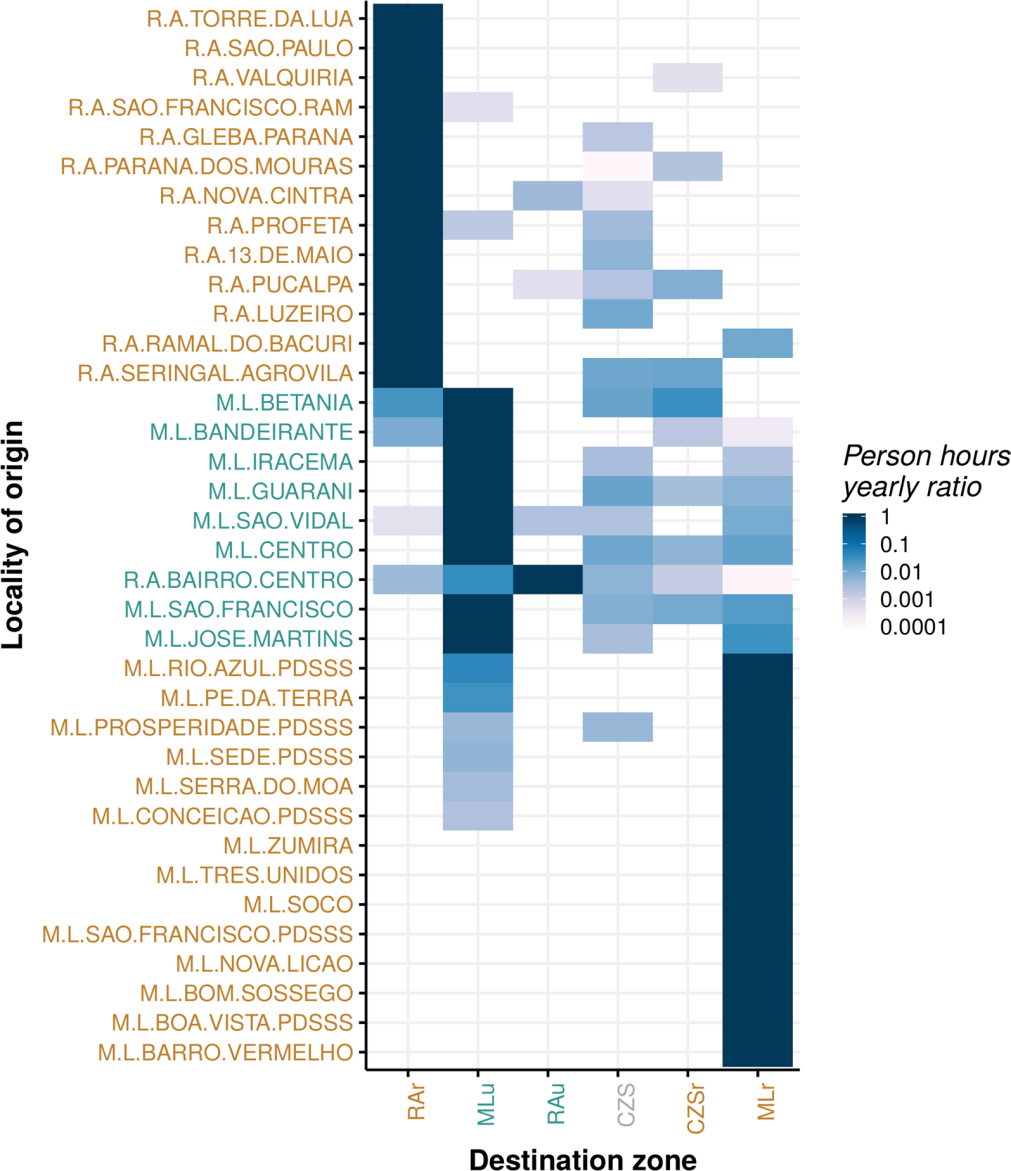
Heatmap of person-hours annual ratio spent in each zone by locality of residence. Color gradient based on the log-scale of the ratio, ranging from white ($<0.0001$) to dark blue (1). Locality (rows) and zone (columns) names are coloured by their type: urban (blue), rural (brown), or nonspecific (gray). Localities’ names have a prefix of M.L or R.A. to indicate the corresponding municipality, ML, or RA. The suffixes u and r in each destination zone indicate urban and rural zones in the corresponding municipality, respectively

In general, respondents exhibited a low rate of displacement to destinations outside their residence zone, with $96\%$ of all households reporting over $90\%$ of their annual person-hour spent at localities within the same zone (Fig. 2). In fact, $93.4\%$ of the households reported at least $90\%$ of person-hours spent in the same residence locality. For reference, a typical Brazilian student will spend at least $10.23\%$ of his/her annual person-hours at school, while a 40 h per week job results in at least about $22\%$ of annual person-hours at work. No household members travelled outside their residence zone for either work or study in $62.2\%$ and $18.6\%$ urban ML and RA households, respectively. Percentages were higher among rural residents, reaching $84.4\%$ and $81.0\%$ for ML and RA, respectively.

### Mobility motivation

**Fig. 3:**
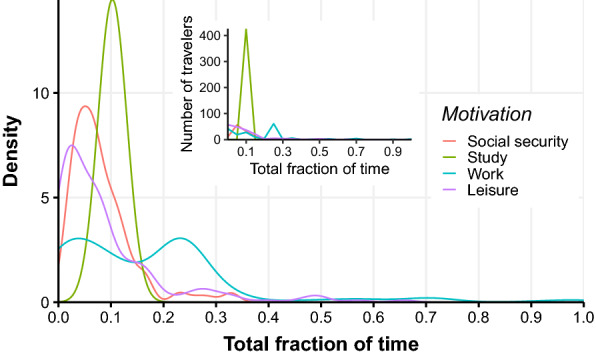
Fraction of time spent in the last 12 months per destination, by respondent. Each curve corresponds to a specific travel motivation: social security (red), study (green), work (blue) and leisure (purple). The main plot represents the kernel density distribution, while the inset shows the histogram with the number of respondents per bin of size 0.05

**Fig. 4:**
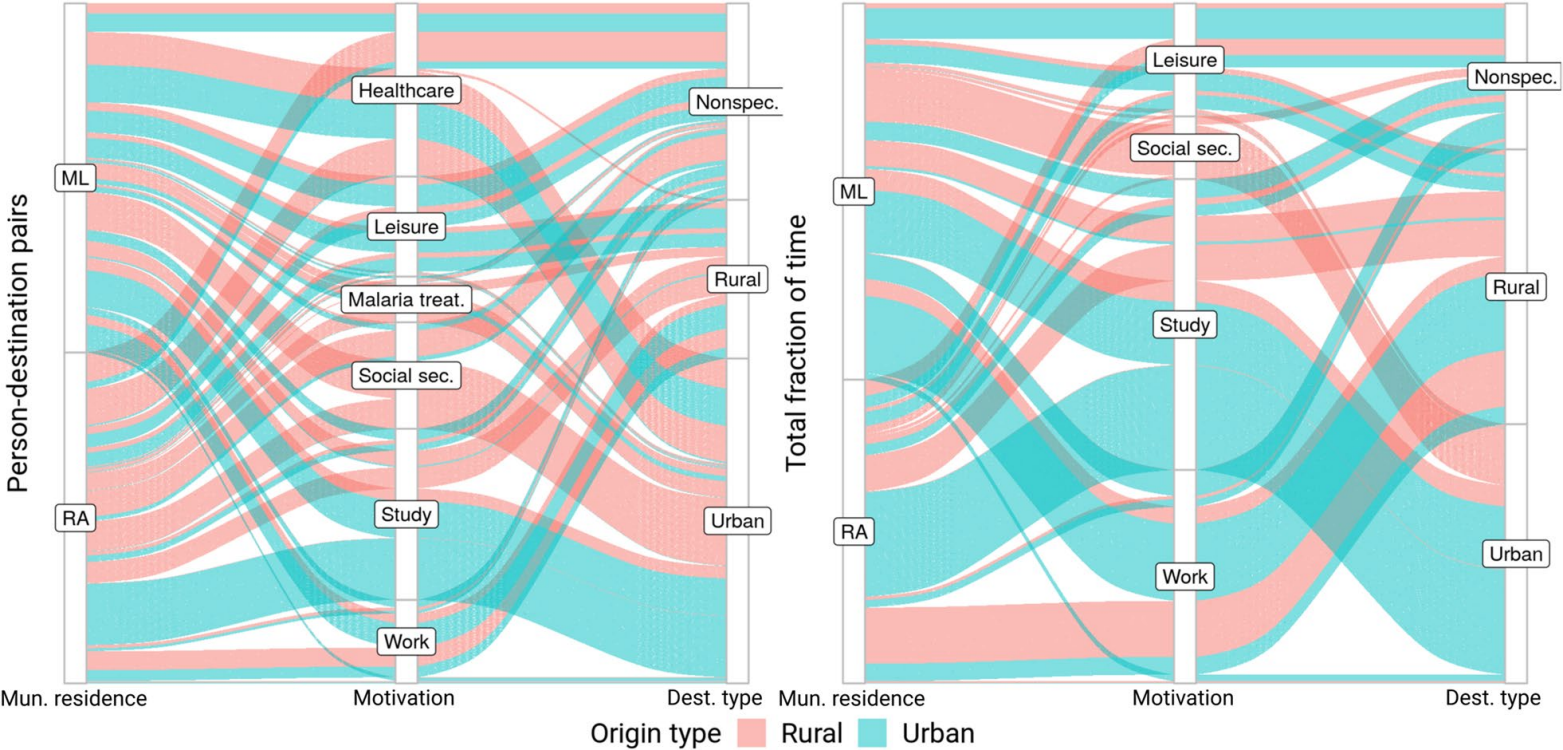
Alluvial diagram of reported displacements. The left panel presents the number of reported destinations per respondent, stratified by municipality and type of origin (red: rural, blue: urban), motivation, and destination type. The right panel presents the total fraction of hours spent in destination per respondent, stratified by municipality and type of origin (red: rural, blue: urban), motivation, and destination type

Figure 3 displays the time distribution spent away from a residence per motivation. The panels in Fig. 4 present the amount of person-destination pairs reported (left panel) and the total fraction of time spent in each destination (right panel). The former provides information on the diversity of destinations, while the latter is essential to assess actual impact in terms of exposure over the 12 month-period. Unfortunately, the time spent on trips motivated by seeking health services was not possible to ascertain.

In terms of person-destination pairs, it is clear that healthcare and study are the main displacement drivers, followed by work, leisure and banking. Participants listed rural localities as their main leisure destinations while referring to urban localities when the motivation was to seek healthcare, study or receive social security benefits. In the case of study-related displacement, the diagram indicates that urban residents are able to remain in urban areas. Concerning rural residents that reported study displacements, the number of residents from ML was similar concerning travellers to urban and rural areas, while those from RA remain in rural areas. On the other hand, individuals that leave their residence for work almost always exhibit rural destinations, regardless of their residence locality.

When taking into account the actual amount of time spent outside their original locality, the main motivations comprise work and study. Although almost no flow from urban to rural areas for study reasons is noted, the opposite is considerable, particularly for ML residents. Conversely, in terms of work displacement, an almost non-observable flow to urban localities is noted, while rural destinations attract residents of both urban and rural areas from both municipalities, with considerable total time spent at the destination. A detailed description of the mobility motivation among respondents is presented in Additional file 1.

### Mobility and malaria

**Fig. 5:**
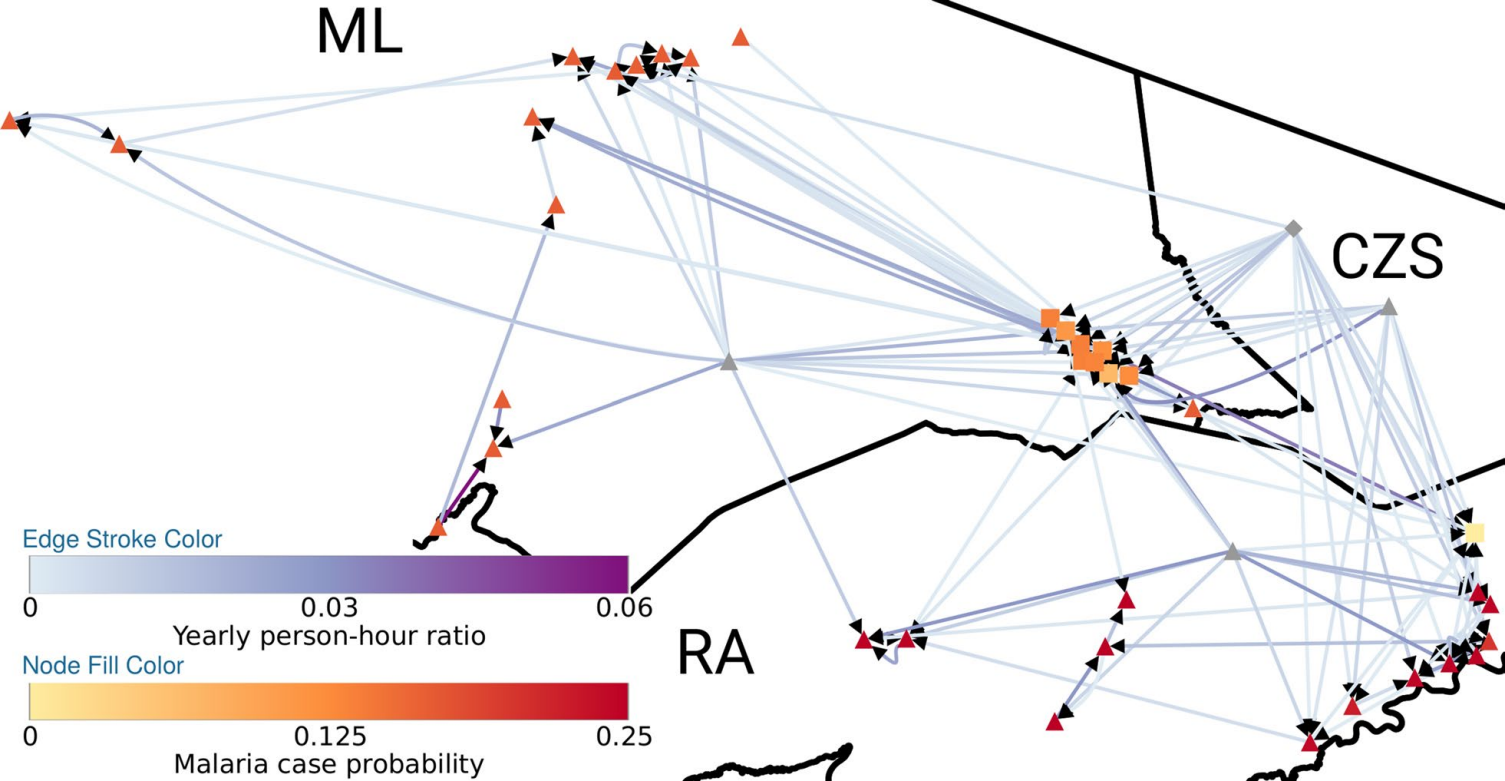
Mobility and malaria probability network. Each node represents a locality surveyed or reported as a place of work/study. Localities outside of the surveyed area were aggregated by municipality (ML, RA, or CZS) and zone type (rural, urban, or nonspecific) and are gray shaded. Nodes’ shape represents rural (triangles), urban (squares), and unspecified (diamonds) zones. Nodes from the surveyed area are displayed according to their geolocation. Nodes representing aggregated localities are displayed in the corresponding municipality. Nodes are coloured according to the estimated malaria case probability based on typical mobility pattern, with a gradient from light yellow (0) to dark red (0.25). Edge’s direction and color reflect the annual person-hours ratio contribution of the locality of edge’s origin to the probability of observing a malaria case at the locality of edge’s destination, with a gradient from light blue (0) to dark purple (0.06). Please refer to the online article for high resolution image

Figure 5 summarizes the effect of travelling on malaria contraction probability at each locality. It is a weighted directed graph (Fig. 5), where nodes represent the localities and directed edges represent the contribution of time spent at the destination on the malaria probability at the origin (arrow). The darker the line color, the more important is the commuting effect on malaria probability at the origin. Nodes are coloured based on the estimated malaria probability using (Eq. 6). It is clear that all localities are well-connected in this network.

A strong connectivity between residence zones is observed. A detailed description of the estimated probability of contracting malaria considering the typical median time spent at each destination is provided in the Additional file 2, since the values reported in the network comprise the medians of the subsequent distribution. This calculation does not differentiate between residing or visiting a locality. The probability is highest in rural RA, followed by rural ML. Urban ML shows a slightly lower risk compared to its urban counterpart. Only urban RA presented a very low malaria contraction probability. Spending time in rural CZS also resulted in a protective effect, albeit not statistically significant (not shown).

### Scenarios

The results of the logistic model are presented in Table 1. The coefficients correspond to the marginal effect of spending $100\%$ of the person-time of a certain household in a rural or urban zone at ML, RA or CZS. These data can be used to estimate the probability of malaria cases in each zone with no displacement. This is computed from the inverse of the logit function (Eq. 6) with ${\bar{\tau }}_{z,z} = 1$, and ${\bar{\tau }}_{z,z'} = 0$ for $z' \ne z$.

**Table 1:** Logistic model results

Zone	Coefficients	Std. error	p value
MLr	$-\,1.28428$	0.10405	< 0.001
MLu	$-\,1.44798$	0.10605	< 0.001
RAr	$-\,0.91538$	0.09384	< 0.001
RAu	$-\,2.52504$	0.18589	< 0.001
CZS	$-\,3.11453$	3.95460	0.4309
CZSr	$-\,16.23128$	7.72548	0.0356

**Fig. 6:**
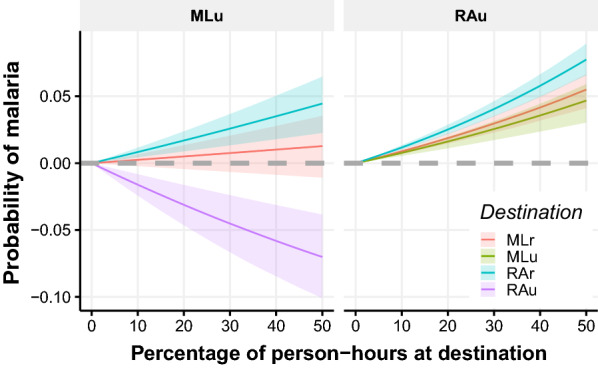
The expected probability of contracting malaria under different commuting scenarios. Contribution in urban areas from ML and RA based on the percentage of person-hours spent at different destination zones. Results obtained from 1000 samples of the posterior distribution of exposure effect of each zone and simulating single-destination mobility profiles

Figure 6 indicates how the probability of contracting malaria for urban resident changes as they spend more time in the other residence zones. Spending person-hours in RAu conferred relative protection to MLu residents. On the other hand, spending time in RAr increased the probability of contracting malaria, although not significant for MLr. For RAu residents, spending time in MLu, MLr and RAr increased the probability of contracting malaria, more so if travelling to the latter.

## Discussion

Mâncio Lima and Rodrigues Alves are important targets for malaria prevention. In 2015, the official system reported a falciparum API of 48 and 64 and vivax API of 294 and 220, respectively, for ML and RA [2]. It was a relatively good year considering the historical trend of these municipalities, but a bad year considering the Amazon Basin as a whole. Risk factors for malaria transmission exist both in urban and rural areas, such as the presence of fish farming, proximity to flooded areas, living at the forest fringe and working in logging activities [9], as well as the few available health teams that must travel to distant places in search of cases. Understanding the mobility of this population can result in new approaches towards malaria control in this region.

The analysis suggests that the Mâncio Lima and Rodrigues Alves populations are not very mobile, with most residents rarely leaving their home area. When they do, most destinations are located within their municipalities. The major commutation hub is CZS, the regional administrative center. Low mobility can be attributed to fuel costs, the scarcity of routes between most rural localities and the difficulty of using the existing routes year round. For example, dirt roads often close during the rainy season while small rivers become too shallow to navigate by larger boats during the dry season [9]. However, it is important to note that some types of commutes, such as those motivated by illegal activities (hunting, drug tracking) may have been omitted by the respondents. Moreover, it is possible that a household survey may have failed to capture truly mobile populations, that is, those without a fixed residence, or those that were away from home during the survey. To capture hidden populations, other study designs are required, including snowball sampling or respondent-driven-sampling.

Malaria cases imports by resident commutation is likely to be low. On the other hand, malaria case exports to other municipalities by ML and RA residents should be monitored. Exports depend on travelling rates and the probability of being infected. Although travelling is infrequent, malaria prevalence is high. Guajará (in the state of Amazonas), and Rio Branco (in the state of Acre) (see Additional file 1) are at risk for malaria imports due to their connectivity with the studied population [2, 5]. Low mobility rates should not be disregarded in a malaria control programme. In such a situation, active traveller testing should be considered to avoid their reinfection, while testing in other Amazon areas is also required. Some studies characterize circulating *Plasmodium* strains, and have demonstrated the importance of long journeys on infection imports [29, 30]. Saita et al. [31] reported a positive association between malaria prevalence during the dry season in the Thai-Myanmar border and road quality, a mobility rate proxy.

The probability of contracting malaria in the Alto Juruá is, overall, more associated with residence area than displacement area, due to low mobility rates. However, some types of commutes result in higher risks than others. It was demonstrated herein that an urban RA resident exhibits an increase of only $5\%$ in the probability of contracting malaria by spending $30\%$ of their time in rural RA, but as high as $50\%$ if spending the same time in ML areas (Fig. 6). The effectiveness of the malaria control programme can be improved by placing testing sites along these routes.

Differences in malaria prevalence along rural-urban gradients can be explained by ecological, social, and behavioural factors [7–9, 31]. In general, urban areas present certain conditions, such as housing, drainage network, pavement and easier access to health care, that contribute to decreased malaria prevalence compared to rural areas [32]. According to Braz et al. [33], a spatial dependency is noted for malaria in the Amazon Basin, with differing characteristics between municipalities, states and international borders, reinforcing the importance of monitoring malaria clusters and integrative control actions. The Alto Juruá, as a whole, is highly receptivity to malaria, since most of the population lives at the forest fringes. This forest is seasonally flooded, creating favourable breeding conditions for the malaria vector [34]. Fish farming also contributes to a high abundance of vectors in both urban and peri-urban areas. Fish-ponds began to be built around 2000 as an important local economic activity. A large fraction of these ponds was completed by 2005 [35], with over 80 fish-ponds built each year [5]. Fish farming contributed to maintaining urban ML as a significant malaria transmission area, despite efforts to remove natural breeding sites. The same was not observed in urban RA, as fish farming conditions are not so suitable.

Studies carried out in Colombia, Peru and Namibia using molecular markers to measure the gene flow between different study areas have suggested the presence of malaria corridors that allow the slow and progressive movement of parasite populations throughout endemic areas, with few evidence of direct human displacement [8, 29, 30, 36]. This is an important issue to consider for the Alto Juruá region when observing prevalence differences throughout urban and rural zones and neighbouring municipalities. The studied localities in rural ML are traditional communities living along the Moa and Azul rivers. Their way of life is mainly based on traditional forest product extractivism and subsistence agriculture. Their mobility is very low and occurs strictly by boat [9]. Conversely, rural RA contains several newly implemented settlements, which means that the new populations were not probably exposed to malaria. Recently arriving in a rural settlement is a known risk for symptomatic malaria [6]. This region comprises more commuting individuals, mainly for work, which follows a season-dependent pattern mostly related to agricultural populations, as reported by Saita et al. [31].

This study exhibits certain limitations. It is a cross-sectional survey, which provides only one measure in time. The interviews were performed as a self-reported questionnaire, with potential memory bias, since events were reported up to 12 months prior to the day of the interview. Another limitation is the fact that the time of day spent in each locality was not accounted for, which can affect the probability for malaria infection, due to vector activity preferences. Seasonality attributed to malaria transmission dynamics may display an important influence in this study, as reported in the literature [3, 31, 32, 34, 37].

## Conclusion

The natural conditions for pathogen circulation, such as *Plasmodium* spp., combined with the Amazon human mobility pattern clearly indicate the need for changes in disease control perspectives. It is paramount that intersectoral public policies be the basis for health mitigation actions. Areas with fish farms must follow adequate policies to support this economic activity, and this policy must comprise the entire process, from the fish-pond concession through maintenance training, as well as the final product destination. This will avoid fish-pond abandonment by owners who are unaware of the malaria-related risks and who are often unable to afford maintenance costs, which is common in the region [38] (personal communication). This is especially true concerning areas close to the forest fringe, as recent settlements have been established and higher active case monitoring is required to improve the opportunity concerning diagnoses and treatment responses, which can, in turn, reduce malaria transmission in these areas. The state of Acre is a reference concerning these protocols and won the Malaria Champion of the Americas award for three consecutive years (2011–2013) [39]. The current issue comprises changes to these policies and investments in Malaria Control Programme. Another important action to consider is to include malaria as a transverse activity in local school curricula, as well as in other Amazon Basin regions. In workshops held with teachers in the region, it was identified that schools focus more on other diseases such as dengue (manuscript in preparation), which results in a higher regional disease burden (submitted manuscript).

## Supplementary information

**Additional file 1.** The table with information about origin-destination pairs by a total of hours, motivation, and type of zone destination and the pdf file with the quantitative description of mobility motivation.

**Additional file 2.** The estimated malaria case probability based on typical person-hour-origin-destination matrix and the estimated effect of each locality to the probability of observing a malaria case. Points represent the median and vertical lines the 95% CI over 1000 samples of the posterior distribution.

**Additional file 3.** The dataset used to conduct the analysis.

## Data Availability

The dataset supporting the conclusions of this article is available in Additional file 3.
