# Supplementary material for: Measuring the contribution of human mobility to malaria persistence
Source: Malar J. 2020 Nov 11;19:404. doi: 10.1186/s12936-020-03474-4 (PMC7659106; doi:10.1186/s12936-020-03474-4)
Supplement: Supplementary file 1 — Additional file 1. The table with information about origin-destination pairs by a total of hours, motivation, and type of zone destination and the pdf file with the quantitative description of mobility motivation. [file 12936_2020_3474_MOESM1_ESM.zip › Addfile1.pdf]

Additional file 1: A detailed description of the mobility motivation among respondents (Household survey in Alto Juruá region, Acre, Brazil)

Regular commutation for studying was reported by 210 (40.5%) households. This was the main reason for commuting. More than 400 individuals (18.9% of sampled individuals and 23.5% of person-destination pairs by motivation) attended educa-tional facilities away from their locality. For these students, the time spent at the destination is ca. 10% of the year or 36.5 person-days, corresponding to 42.7% ofthe total person-hours spent out of household locality. The second most common reason for traveling is work, reported by 153 (29.5%) households. This motivation accounted for 208 person-destination pairs (12.2% of person-destination pairs by motivation), representing 31.3% of the total person-hours spent away from their home locality. The time spent at the workplace presented roughly a bimodal distribution, with a group spending ca. 2.5% of the year at the destination (9 person-days) and another group spending ca. 25% of their time (90 person-day).

Rural households reported 85 person-destination pairs for workrelated travel (from 62 out of 237 households), with 71 of those pairs having rural areas as destination, 5 with urban destinations, and 9 with unspecified destination zone. For urban households, there were a total of 123 person destination pairs forwork (91 out of 282 households), with 82 of those pairs having rural destinations,10 to urban destinations, and 25 to destinations with unspecified zones.

Urban householders will predominantly go to rural areas for work related reasons(71.5% of its work-related person-destination pairs), while commuting to other ur-ban localities for study (83.9% of study-related person-destination pairs). On the other hand, rural householders that need to leave their locality for either work or study would mostly go to rural localities, with 83.5% of work-related and 67.9% of study-related person-destination pairs with rural destinations, respectively.

From the total of 519 households, 292 (56.3%) reported displacement to another locality to seek health care assistance. From this total, 92 households (17.7%) reported displacement specifically for malaria treatment. In general, health relatedtrips were more frequent among rural than urban households (178/237 and 114/282,respectively). For rural households, 78.7% of them reported at least one trip to ur-ban localities. Conversely, there were no health related trips from urban households to rural localities reported. While reported health related trips from ML were al-most all within the same municipality (159/229 household-destination pairs), fromthe 148 household-destination pairs originated

in RA, most were distributed between RA (38.5%) and CZS (48.7%) localities. We could not calculate the time spent away for health related activities, since this information was not available.

A smaller fraction of trips was motivated by seeking urban services (mainly bank-ing for retrieving social security benefits). This was reported by 238 (45.9%) house-holds, most of them living in the rural areas. For rural households inML, thedestination was almost always localities in ML itself (94/100 household-destination pairs), while for those in RA the destination was again distributed between localities in RA (60/139) and CZS (65/139). A total of 265 (15.6%) person-destination pairs were driven by this necessity, but accounted for just 9.2% of the total time spent outside home. The median time dedicated to this activity was 9 person-day per year per destination (2.5% of the total time).

Displacements for leisure accounted for 249 (14.6%) of the trips, representing16.6% of the total amount of time spent out of household locality. The median timespent at each destination corresponded to 5.8% of the householder’s year (ca. 18 person-day). For rural households, most household-destination pairs were to CZS (20/74), Rio Branco (19/74), capital of Acre state, ML (18/74), and RA (9/74) localities. For urban ones, most pairs were to Rio Branco (58/175), ML (43/175), RA (27/175), and CZS (23/175). There were 15 (2.9%) trips for miscellaneous motivations.
