# Supplementary figures and images for: Measuring the contribution of human mobility to malaria persistence
Source: Malar J. 2020 Nov 11;19:404. doi: 10.1186/s12936-020-03474-4 (PMC7659106; doi:10.1186/s12936-020-03474-4)

**Malaria case probability**

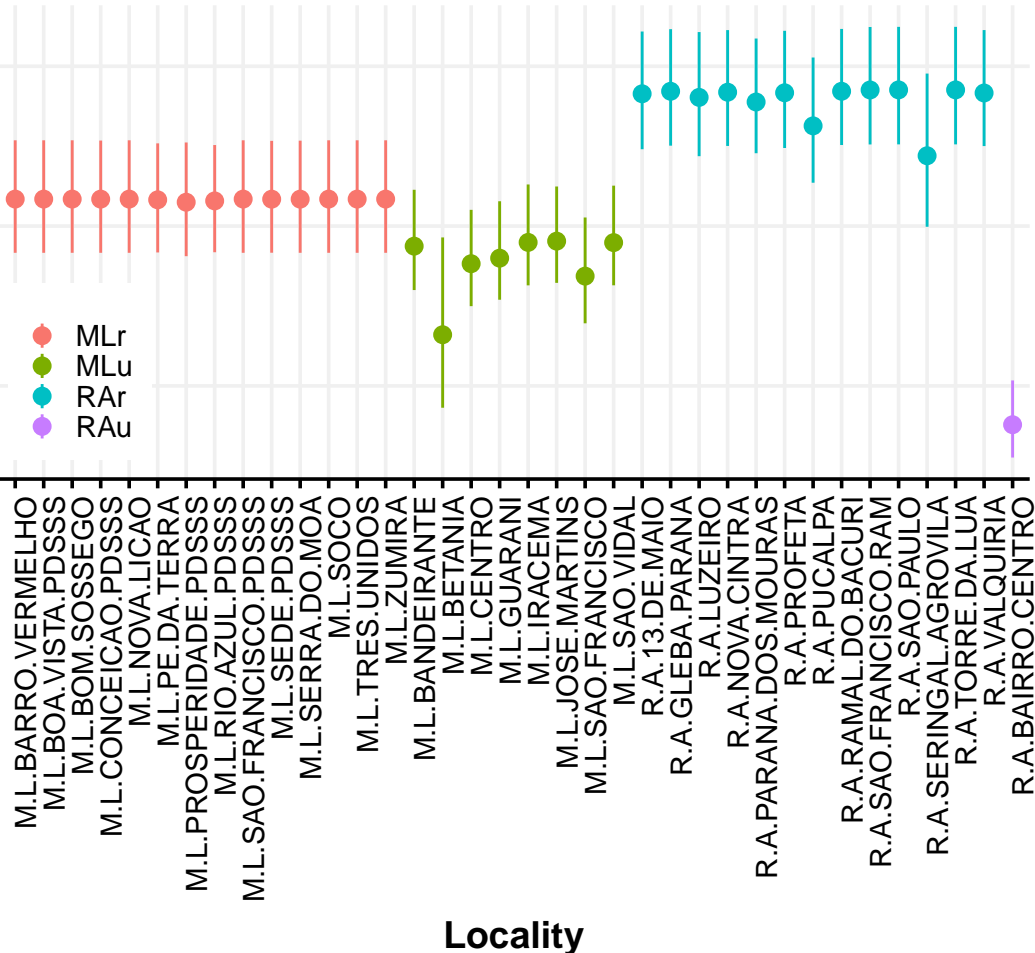

Supplement: Supplementary file 2 — Additional file 2. The estimated malaria case probability based on typical person-hour-origin-destination matrix and the estimated effect of each locality to the probability of observing a malaria case. Points represent the median and vertical lines the 95% CI over 1000 samples of the posterior distribution. [file 12936_2020_3474_MOESM2_ESM.pdf]
